# Genome sequence of the clover-nodulating *Rhizobium leguminosarum* bv. *trifolii* strain TA1

**DOI:** 10.4056/sigs.4488254

**Published:** 2013-12-15

**Authors:** Wayne Reeve, Rui Tian, Sofie De Meyer, Vanessa Melino, Jason Terpolilli, Julie Ardley, Ravi Tiwari, John Howieson, Ronald Yates, Graham O’Hara, Mohamed Ninawi, Hazuki Teshima, David Bruce, Chris Detter, Roxanne Tapia, Cliff Han, Chia-Lin Wei, Marcel Huntemann, James Han, I-Min Chen, Konstantinos Mavromatis, Victor Markowitz, Natalia Ivanova, Galina Ovchinnikova, Ioanna Pagani, Amrita Pati, Lynne Goodwin, Sam Pitluck, Tanja Woyke, Nikos Kyrpides

**Affiliations:** 1 Centre for Rhizobium Studies, Murdoch University, Western Australia, Australia; 2 Department of Agriculture and Food, Western Australia, Australia; 3 DOE Joint Genome Institute, Walnut Creek, California, USA; 4 Los Alamos National Laboratory, Bioscience Division, Los Alamos, New Mexico, USA; 5 Biological Data Management and Technology Center, Lawrence Berkeley National Laboratory, Berkeley, California, USA

**Keywords:** root-nodule bacteria, nitrogen fixation, rhizobia, *Alphaproteobacteria*

## Abstract

*Rhizobium leguminosarum* bv. *trifolii* strain TA1 is an aerobic, motile, Gram-negative, non-spore-forming rod that is an effective nitrogen fixing microsymbiont on the perennial clovers originating from Europe and the Mediterranean basin. TA1 however is ineffective with many annual and perennial clovers originating from Africa and America. Here we describe the features of *R. leguminosarum* bv. *trifolii* strain TA1, together with genome sequence information and annotation. The 8,618,824 bp high-quality-draft genome is arranged in a 6 scaffold of 32 contigs, contains 8,493 protein-coding genes and 83 RNA-only encoding genes, and is one of 20 rhizobial genomes sequenced as part of the DOE Joint Genome Institute 2010 Community Sequencing Program.

## Introduction

Biological fixation of inert atmospheric dinitrogen gas is a process that can only be performed by certain prokaryotes in the domains *Archaea* and *Bacteria.* By far the greatest amounts of nitrogen (N) are fixed by specialized soil bacteria (root nodule bacteria or rhizobia) that form proto-cooperative, non-obligatory symbiotic relationships with legumes [1]. Indeed, these symbioses contribute ~40 million tonnes of N annually to support global food production [2].

Species of the legume genus *Trifolium* (clovers) are amongst the most widely cultivated pasture legumes. Naturally, this genus inhabits three distinct centers of diversity with approximately 28% of species in the Americas, 57% in Eurasia and 15% in Sub-Saharan Africa [3]. A smaller subset of about 30 species, almost all of Eurasian origin, are widely gown as annual and perennial species in pasture systems in Mediterranean and temperate regions [3]. Globally important perennial species of clover include *T. repens* (white clover), *T. pratense* (red clover), *T. fragiferum* (strawberry clover) and *T. hybridum* (alsike clover). Clovers usually form N_2_-fixing symbioses with the common soil bacterium *Rhizobium leguminosarum* bv. *trifolii,* and different combinations of *Trifolium* hosts and strains of *R. leguminosarum* bv. *trifolii* can vary markedly in symbiotic compatibility [4], resulting in a broad range of symbiotic developmental outcomes ranging from ineffective (non-nitrogen fixing) nodulation to fully effective N_2_-fixing partnerships [5].

In Australia, *Rhizobium leguminosarum* bv. *trifolii* strain TA1 (initially designated BA-Tas) has a long history of use as a commercial inoculant for *Trifolium* spp. [6]. TA1 was originally isolated from a root nodule on the annual species *T. subterraneaum* in Bridport, Tasmania in the early 1950’s [6]. This isolate is likely to be a naturalized strain of European origin that arrived by chance in Tasmania in the 1800’s. Although widely used as a microsymbiont of European clovers, it became evident that this soil saprophyte is not acid tolerant [7] and survives poorly when coated onto clover seed with a peat based carrier [8-10]. Nevertheless, TA1 remains the commercial inoculant in Australia for perennial (*T repens*, *T. pratense, T. fragiferum*, *T. hybridum*, *T. tumens* (talish clover)) and annual (*T. alexandrinum* (berseem clover), *T. glomeratum* (cluster clover) and *T. dubium* (suckling clover)) clovers of European origin [11]. Furthermore, this *R. leguminosarum* bv. *trifolii* strain has been adopted by the international community as a model organism to investigate the biology of the *Trifolium-Rhizobium* symbiosis [12]. Here we present a summary classification and a set of general features for *R. leguminosarum* bv. *trifolii* strain TA1 together with the description of the complete genome sequence and its annotation.

## Classification and general features

**Figure 1:**
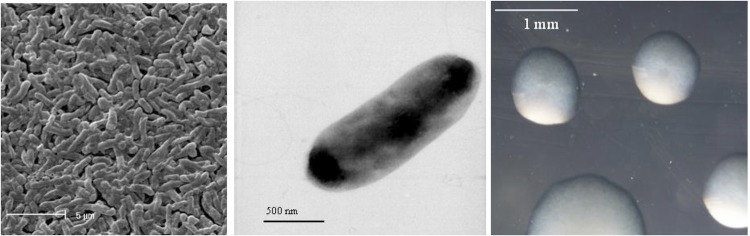
Images of *Rhizobium leguminosarum* bv. *trifolii* strain TA1 using scanning (Left) and transmission (Center) electron microscopy as well as light microscopy to visualize colony morphology on solid media (Right).

**Figure 2:**
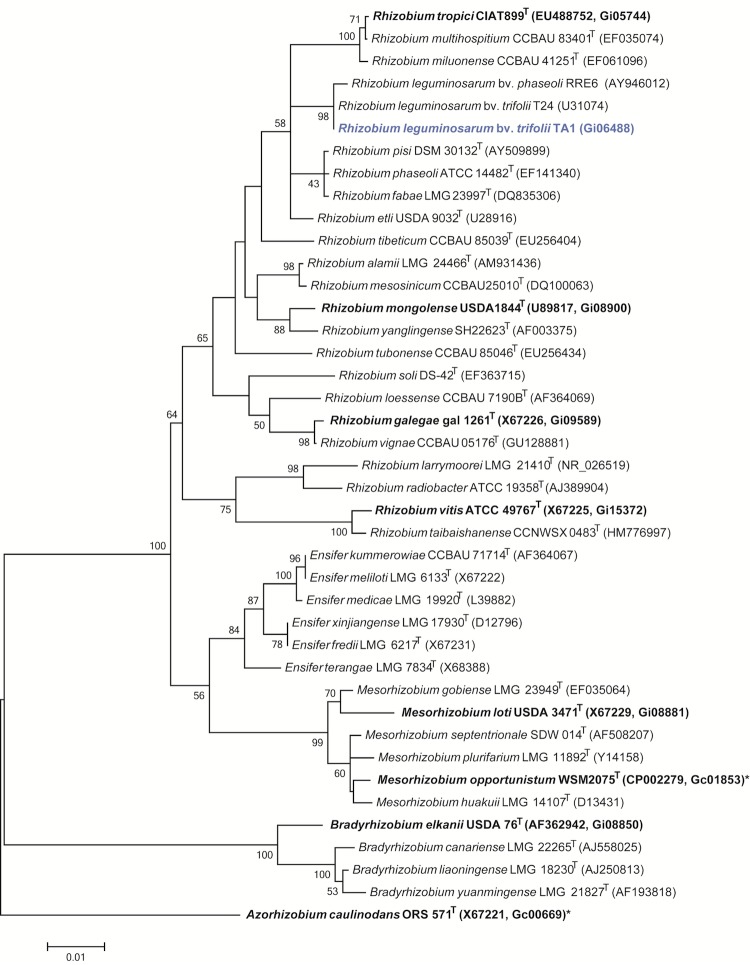
Phylogenetic tree showing the relationship of *Rhizobium leguminosarum* bv. *trifolii* strain TA1 (shown in blue print) with some of the root nodule bacteria in the order *Rhizobiales* based on aligned sequences of the 16S rRNA gene (1,307 bp internal region). All sites were informative and there were no gap-containing sites. Phylogenetic analyses were performed using MEGA, version 5.05 [31]. The tree was built using the maximum likelihood method with the General Time Reversible model. Bootstrap analysis [32] with 500 replicates was performed to assess the support of the clusters. Type strains are indicated with a superscript T. Strains with a genome sequencing project registered in GOLD [33] are in bold print and the GOLD ID is mentioned after the accession number. Published genomes are designated with an asterisk.

*R. leguminosarum* bv. *trifolii* strain TA1 is a motile, Gram-negative, non-spore-forming rod (Figure 1 Left and Center) in the order *Rhizobiales* of the class *Alphaproteobacteria*. It is slow growing, forming 1-4 mm diameter colonies within 3-5 days grown on half Lupin Agar (½LA) [13] at 28°C. Colonies on ½LA are white-opaque, slightly domed, moderately mucoid with smooth margins (Figure 1 Right). Minimum Information about the Genome Sequence (MIGS) is provided in Table 1. Figure 2 shows the phylogenetic neighborhood of *R. leguminosarum* bv. *trifolii* strain TA1 in a 16S rRNA sequence based tree. This strain clusters closest to *R. leguminosarum* bv. *trifolii* T24 and *R. leguminosarum* bv. *phaseoli* RRE6 with 99.9% and 99.8% sequence identity, respectively.

**Table 1:** Classification and general features of *Rhizobium leguminosarum* bv. *trifolii* strain TA1 according to the MIGS recommendations [14].

**MIGS ID**	**Property**	**Term**	**Evidence code**
	Current classification	Domain *Bacteria*	TAS [15]
		Phylum *Proteobacteria*	TAS [16]
		Class *Alphaproteobacteria*	TAS [17,18]
		Order *Rhizobiales*	TAS [17,19]
		Family *Rhizobiaceae*	TAS [20,21]
		Genus *Rhizobium*	TAS [20,22-25]
		Species *Rhizobium leguminosarum* bv. *trifolii*	TAS [20,22,25,26]
	
	Gram stain	Negative	TAS [27]
	Cell shape	Rod	TAS [27]
	Motility	Motile	TAS [27]
	Sporulation	Non-sporulating	TAS [27]
	Temperature range	Mesophile	TAS [27]
	Optimum temperature	28°C	TAS [27]
	Salinity	Not reported	
MIGS-22	Oxygen requirement	Aerobic	TAS [27]
	Carbon source	Varied	
	Energy source	Chemoorganotroph	TAS [27]
MIGS-6	Habitat	Soil, root nodule, on host	IDA
MIGS-15	Biotic relationship	Free living, symbiotic	IDA
MIGS-14	Pathogenicity	Non-pathogenic	TAS [27]
	Biosafety level	1	TAS [28]
	Isolation	Root nodule of *Trifolium subterraneum*	TAS [29]
MIGS-4	Geographic location	Bridport, Tasmania	IDA
MIGS-5	Nodule collection date	1953	IDA
MIGS-4.1	Longitude	147.667	IDA
MIGS-4.2	Latitude	-41.0335	IDA
MIGS-4.3	Depth	Not recorded	
MIGS-4.4	Altitude	Not recorded	

Evidence codes – IDA: Inferred from Direct Assay; TAS: Traceable Author Statement (i.e., a direct report exists in the literature). These evidence codes are from the Gene Ontology project [30].

### Symbiotaxonomy

*Rhizobium leguminosarum* bv. *trifolii* strain TA1 is currently the commercial inoculant for white (*Trifolium repens*), red (*Trifolium pratense*) and strawberry (*Trifolium fragiferum*) clovers in Australia. TA1 in general is not as effective for nitrogen fixation on annual clovers as other strains, such as WSM1325 [34,35]. However TA1 is of particular interest because it displays a broad host range for nodulation and nitrogen fixation across annual and perennial clovers originating from the European and Mediterranean centre of origin of clovers [1]. TA1 is generally able to nodulate but unable to fix with many annual and and perennial clovers originating from Africa and America [34].

## Genome sequencing and annotation information

### Genome project history

This organism was selected for sequencing on the basis of its environmental and agricultural relevance to issues in global carbon cycling, alternative energy production, and biogeochemical importance, and is part of the Community Sequencing Program at the U.S. Department of Energy, Joint Genome Institute (JGI) for projects of relevance to agency missions. The genome project is deposited in the Genomes OnLine Database [33] and an improved-high-quality-draft genome sequence in IMG. Sequencing, finishing and annotation were performed by the JGI. A summary of the project information is shown in Table 2.

**Table 2:** Genome sequencing project information for *Rhizobium leguminosarum* bv. *trifolii* strain TA1.

**MIGS ID**	**Property**	**Term**
MIGS-31	Finishing quality	Improved high-quality draft
MIGS-28	Libraries used	Illumina GAii shotgun and paired end 454 libraries
MIGS-29	Sequencing platforms	Illumina GAii and 454 GS FLX Titanium technologies
MIGS-31.2	Sequencing coverage	7.8× 454 paired end, 764.2× Illumina
MIGS-30	Assemblers	Velvet 1.0.13, Newbler 2.3, phrap 4.24
MIGS-32	Gene calling methods	Prodigal 1.4, GenePRIMP
	GOLD ID	Gi0648
	NCBI project ID	63831
	Database: IMG	2510461076
	Project relevance	Symbiotic N_2_ fixation, agriculture

### Growth conditions and DNA isolation

*Rhizobium leguminosarum* bv. *trifolii* strain TA1 was grown to mid logarithmic phase in TY rich media [36] on a gyratory shaker at 28°C. DNA was isolated from 60 ml of cells using a CTAB (Cetyl trimethyl ammonium bromide) bacterial genomic DNA isolation method [37].

### Genome sequencing and assembly

The genome of *Rhizobium leguminosarum* bv. *trifolii* strain TA1 was sequenced at the Joint Genome Institute (JGI) using a combination of Illumina [38] and 454 technologies [39]. An Illumina GAii shotgun library which generated 66,421,308 reads totaling 5,048 Mb, and a paired end 454 library with an average insert size of 13 kb which generated 393,147 reads totaling 100.1 Mb of 454 data were generated for this genome. All general aspects of library construction and sequencing performed at the JGI can be found at the JGI user homepage [40]. The initial draft assembly contained 199 contigs in 5 scaffolds. The 454 paired end data was assembled with Newbler, version 2.3. The Newbler consensus sequences were computationally shredded into 2 kb overlapping fake reads (shreds). Illumina sequencing data were assembled with VELVET, version 1.0.13 [41], and the consensus sequence were computationally shredded into 1.5 kb overlapping fake reads (shreds). We integrated the 454 Newbler consensus shreds, the Illumina VELVET consensus shreds and the read pairs in the 454 paired end library using parallel phrap, version SPS - 4.24 (High Performance Software, LLC). The software Consed [42-44] was used in the following finishing process. Illumina data was used to correct potential base errors and increase consensus quality using the software Polisher developed at JGI (Alla Lapidus, unpublished). Possible mis-assemblies were corrected using gapResolution (Cliff Han, unpublished), Dupfinisher (Han, 2006), or sequencing cloned bridging PCR fragments with subcloning. Gaps between contigs were closed by editing in Consed, by PCR and by Bubble PCR (J-F Cheng, unpublished) primer walks. A total of 275 additional reactions were necessary to close gaps and to raise the quality of the finished sequence. The estimated genome size is 7.6 Mb and the final assembly is based on 65.3 Mb of 454 draft data which provides an average of 8.6× coverage of the genome and 4,864.7 Mb of Illumina draft data which provides an average 640.1× coverage of the genome.

### Genome annotation

Genes were identified using Prodigal [45] as part of the DOE-JGI Annotation pipeline [46], followed by a round of manual curation using the JGI GenePRIMP pipeline [47]. The predicted CDSs were translated and used to search the National Center for Biotechnology Information (NCBI) non-redundant database, UniProt, TIGRFam, Pfam, PRIAM, KEGG, COG, and InterPro databases. These data sources were combined to assert a product description for each predicted protein. Non-coding genes and miscellaneous features were predicted using tRNAscan-SE [48], RNAMMer [49], Rfam [50], TMHMM [51], and SignalP [52]. Additional gene prediction analyses and functional annotation were performed within the Integrated Microbial Genomes (IMG-ER) platform [37,53].

## Genome properties

**Figure 3:**
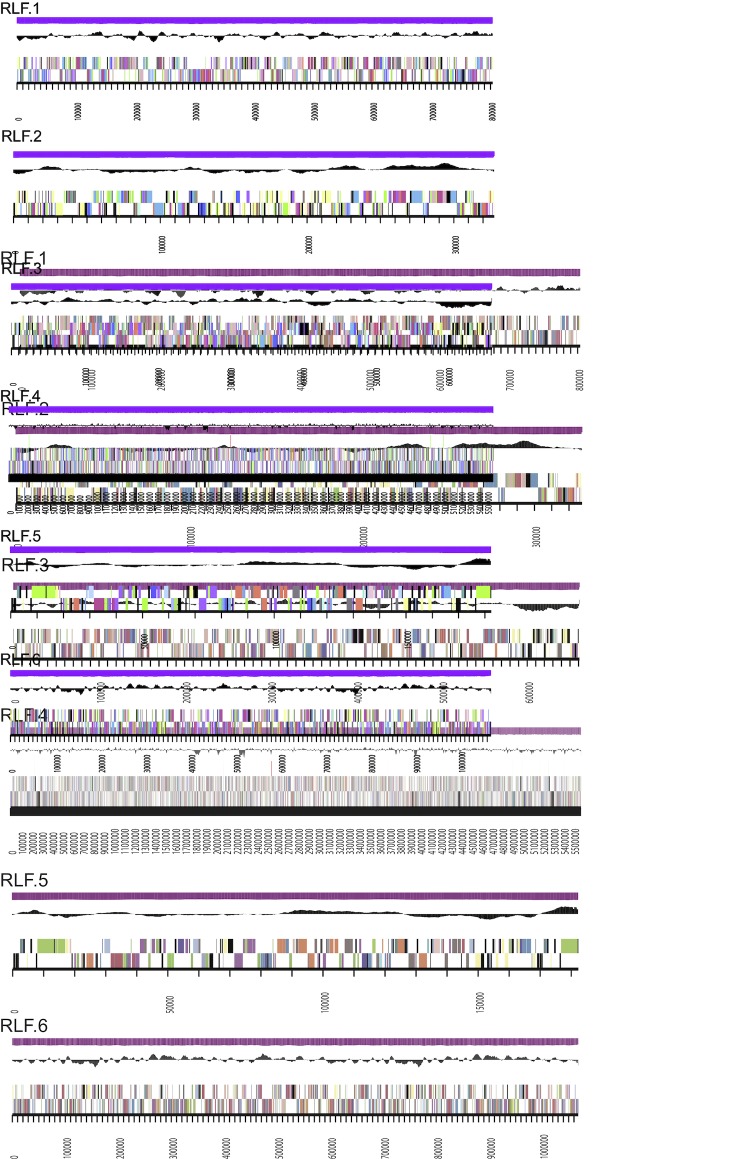
Graphical linear map of the genome of *Rhizobium leguminosarum* bv. *trifolii* strain TA1. From outside to the center: Genes on forward strand (color by COG categories), Genes on reverse strand (color by COG categories), RNA genes (tRNAs green, sRNAs red, other RNAs black), GC content, GC skew.

The genome is 8,618,824 nucleotides with 60.74% GC content (Table 3) and comprised of 32 contigs in 6 scaffolds (Figure 3). From a total of 8,576 genes, 8,493 were protein encoding and 83 RNA only encoding genes. The majority of genes (77.85%) were assigned a putative function whilst the remaining genes were annotated as hypothetical. The distribution of genes into COGs functional categories is presented in Table 4.

**Table 3:** Genome sequencing project information for *Rhizobium leguminosarum* bv. *trifolii* strain SRDI943.

**Attribute**	**Value**	**% of Total**
Genome size (bp)	8,618,824	100.00
DNA coding region (bp)	7,407,820	85.95
DNA G+C content (bp)	5,234,677	60.74
Number of scaffolds	6	
Number of contigs	32	
Total genes	8,576	100.00
RNA genes	83	0.97
rRNA operons*	1	0.01
Protein-coding genes	8,493	99.03
Genes with function prediction	6,676	77.85
Genes assigned to COGs	6,673	77.81
Genes assigned Pfam domains	6,944	80.97
Genes with signal peptides	727	8.48
Genes with transmembrane helices	1,897	22.12
CRISPR repeats	0	

*1 copy of 23S rRNA, 2 copies of 16S and 2 copies of 5S rRNA genes

**Table 4:** Number of protein coding genes of *Rhizobium leguminosarum* bv. *trifolii* TA1 associated with the general COG functional categories.

Code	Value	%age	COG Category
J	247	3.29	Translation, ribosomal structure and biogenesis
A	1	0.01	RNA processing and modification
K	751	10.01	Transcription
L	317	4.23	Replication, recombination and repair
B	3	0.04	Chromatin structure and dynamics
D	44	0.59	Cell cycle control, mitosis and meiosis
Y	0	0.00	Nuclear structure
V	92	1.23	Defense mechanisms
T	402	5.36	Signal transduction mechanisms
M	365	4.87	Cell wall/membrane biogenesis
N	100	1.33	Cell motility
Z	2	0.03	Cytoskeleton
W	0	0.00	Extracellular structures
U	114	1.52	Intracellular trafficking and secretion
O	217	2.89	Posttranslational modification, protein turnover, chaperones
C	384	5.12	Energy production conversion
G	746	9.95	Carbohydrate transport and metabolism
E	803	10.71	Amino acid transport metabolism
F	134	1.79	Nucleotide transport and metabolism
H	235	3.13	Coenzyme transport and metabolism
I	271	3.61	Lipid transport and metabolism
P	374	4.99	Inorganic ion transport and metabolism
Q	201	2.68	Secondary metabolite biosynthesis, transport and catabolism
R	976	13.02	General function prediction only
S	720	9.60	Function unknown
-	1,903	22.19	Not in COGS
